# Should We Really Be Afraid of “Weakness”? Applying the Insights of Attribution Theory

**DOI:** 10.1177/00332941241231210

**Published:** 2024-02-27

**Authors:** Adam Abdulla

**Affiliations:** 1018 School of Applied Social Studies, Robert Gordon University, Aberdeen, UK

**Keywords:** Weakness, area for improvement, expectancy, perceived self-efficacy, attribution theory, stability, controllability, internality

## Abstract

It is widely assumed that the term “weakness” has negative psychological effects and should be replaced by “area for improvement.” The present study is the first to examine the matter experimentally. It was hypothesised that effects of “weakness” (vs. “area for improvement”) are most pronounced in those with low perceived self-efficacy in the relevant domain. Two experiments were conducted in the domain of self-regulation. In those with low perceived self-efficacy for self-regulation (PSESR), “weakness” apparently had a negative indirect effect on improvement expectancy by increasing the perceived stability (Experiment 1) or lowering the perceived controllability (Experiment 2) of the problem. Moreover, at low levels of PSESR in Experiment 2, estimated indirect effects of “weakness” on perceived value of improvement were both positive and negative. However, gender apparently moderated those effects. “Weakness” apparently lowered perceived controllability in both males and females but in women the negative effect was more pronounced when PSESR was low. In addition, “weakness” apparently increased perceived internality in males with low PSESR. Compared to “area for improvement,” “weakness” may indeed have some (negative) psychological effects in people with low perceived self-efficacy in the relevant domain. Given the ubiquity of these terms in evaluative contexts and the widespread fears of the term “weakness,” more experimental research needs to be conducted.

“I refuse to use the word weakness when talking about athletes…” (Connolly & White, 2017, p. 190)

“As this is a strengths model, the term weakness is not used” (Sullivan, 2018, p. 237, italics added)

“As someone who doesn’t hand out grades but rather assesses and has feedback discussions with students, I shudder at the word “weakness”” (Ripp, 2012, italics added)

## “Weakness” Versus “Area for Improvement” and Self-Regulation

Imagine an individual – Ben – who struggles with self-regulation. Ben tends to procrastinate, leaving important work until the last minute. In many settings, Ben would be encouraged to think of this tendency as a “weakness.” However, there is a widespread aversion to the term “weakness,” as indicated by the quotes above. Connolly and White (2017, p. 190), for example, argue that “weakness” implies “an *inherent* deficiency that *cannot be changed or controlled*” (italics added). Other critics argue that use of the term “weakness” lowers people’s hopes of improvement (e.g. Life at United World, 2013).

As will be explained later in the introduction, the case against “weakness” can be expressed in terms from Weiner’s attribution theory (e.g. Weiner, 1985). According to critics, “weakness” increases the perceived *internality* and *stability* of a problem’s cause, whilst lowering its perceived *controllability* and reducing *expectancy* of improvement (e.g. Boroson, 2011; Rael, 2012; Ripp, 2012; Spinella, 2020).

If “weakness” does indeed lower expectancy of improvement (by increasing the perceived stability (PS) or lowering the perceived controllability (PC) of a problem’s cause), the consequences may be severe. Expectancy is one of the primary determinants of motivation or commitment (e.g. Klein & Wright, 1994; Weiner, 1985; Wigfield & Eccles, 2000). Thus if “weakness” has a negative effect on expectancy, it may reduce people’s motivation to improve. Another major determinant of motivation is *perceived value.* If “weakness” also lowers the *perceived value* of improvement (PVOI), it may further undermine motivation.

Perhaps the most commonly proposed alternative to “weakness” is “area for improvement” (e.g. Adair, 2004; DuBrin, 2018; Grensing-Pophal, 1999; Hansten & Jackson, 2009; Kandola et al., 2001; Logan & Royse, 2010; Pahomov, 2014; Tapper, 2014). One source recommends: “Use the phrase “area of improvement”…instead of “weakness,” as the word “weakness” implies that it is static and can’t be changed” (Interview Skills Workbook, nd). According to such sources, an expression such as “area for improvement” does not have the negative connotations of stability and uncontrollability that are apparently associated with “weakness.”

The terms “weakness” and “area for improvement” are both ubiquitous. They are used on a daily basis with millions of individuals around the English-speaking world in self-assessments, performance appraisals, teacher feedback, progress reports, coaching conversations and many other situations. Understanding their psychological effect is therefore of the utmost importance. Given the frequency with which these terms are used, it may be assumed that a great deal of research has compared them. Experimental studies in other domains have compared the effects of different words, for example “challenging” versus “monotonous” (Tolin, 1970), “allow” versus “forbid” (Gesser-Edelsbury et al., 2015), and “wife” versus “partner” (Strunk & Bailey, 2015). Surprisingly, however, no published experimental research has examined “weakness” versus “area for improvement.” Recommendations to replace the former with the latter therefore lack empirical support.

The present study is (to the author’s knowledge) the first to compare the effects of the two expressions in randomised controlled experiments. The conceptual framework for the study was Weiner’s attribution theory (e.g. Weiner, 1985, 2012) and the focal domain was self-regulation. Self-regulation includes managing one’s time, avoiding distractions and overcoming the urge to procrastinate. It has been said that “nearly every major personal and social problem affecting large numbers of modern citizens involves some kind of failure of self-regulation” (Vohs & Baumeister, 2004, p. 3). Examining the effects of “weakness” and “area for improvement” in the domain of self-regulation is therefore extremely important.

The present study focused specifically on self-regulation in learning. In order to be successful as learners, individuals must set goals, plan their work, manage their time and ignore distractions (e.g. Zimmerman, 2002). Self-regulation in learning is essential not only for students in education but also for those in the workplace. As Bell (2017, p. 117) points out, “employees are increasingly being asked to engage in self-directed learning.” Beliefs about one’s capacity to self-regulate in learning are associated with motivation and achievement (Usher & Pajares, 2008). Moreover, self-regulation in learning is consistently related to achievement (e.g. Michaelides & Durkee, 2021; Zimmerman & Kitsantas, 2014). If “weakness” negatively affects attitudes towards self-regulation, both motivation and achievement may suffer.

A simple research question for the present study might be as follows: when individuals are asked to reflect on an unsatisfactory aspect of their own self-regulation, what are the effects of the expressions “weakness” and “area for improvement” on perceived stability, perceived controllability, perceived internality, improvement expectancy and perceived value of improvement? However, one of the key hypotheses of the present study was that the relative effects of these terms *depend on perceived self-efficacy and gender*.

The rest of the introduction is organised as follows. First, Weiner’s attribution theory is introduced and combined with research on mindsets to explain how “weakness” may affect improvement expectancy via perceived stability and controllability. Second, possible effects of “weakness” on perceived value of improvement are considered. Third, reasons for considering perceived self-efficacy (PSE) and gender as potential moderators of effects are presented. Finally, the hypotheses for the present study are recapitulated.

## “Weakness,” “Area for Improvement,” Attribution Theory and Mindsets

Weiner has argued that causes of success or failure have three properties: (i) *perceived stability*, (ii) *perceived controllability* and (iii) *perceived locus,* i.e. *internality* or *externality* (e.g. Weiner, 1985, 2012). In other words, a problem may be attributed to a cause that is (i) stable or unstable over time, (ii) controllable or uncontrollable and (iii) internal (i.e. inside the person) or external (i.e. outside the person).

Weiner contends that the perceived stability of a problem’s cause affects *expectancy* of success or improvement (e.g. Weiner, 1985). Others argue that perceived controllability also influences expectancy (e.g. Forsyth & McMillan, 1981). When individuals attribute difficulties to stable/uncontrollable factors, they do often report lower expectancy of improvement (Kovenklioglu & Greenhaus, 1978; McMahan, 1973; Rudisill, 1988). Weiner argues that perceived locus (i.e. whether the cause of a problem is perceived to be internal or external) does not directly affect expectancy but does influence positive and negative affect (e.g. Weiner, 2012).

Informing individuals that they have a “weakness” may lead them to attribute a problem to a stable, uncontrollable and internal cause. Indeed, this is precisely what many critics actually claim (e.g. Connolly & White, 2017). On the other hand, the expression “area for improvement” implies that the area *may be improved*, which suggests that the problem is less stable and more controllable. In addition, if an issue is described as an “area for improvement” (rather than a “weakness”) individuals may be less likely to conclude that there is a problem *within them*. In short, “weakness” and “area for improvement” may have contrasting effects on perceived stability, controllability and locus (internality).

“Weakness” and “area for improvement” may also lead to differences in perceived stability, controllability and locus by activating a “fixed” versus “incremental” *mindset*. Research on mindset and implicit views of the self has shown that particular attributions may be triggered by written communication. According to “fixed” theories of the self, traits such as ability are permanent and unalterable. According to an “incremental” theory such traits are malleable and may be improved. Hong et al. (1999) found that a fixed/incremental theory could be induced in participants by a few sentences endorsing the relevant theory. Furthermore, participants exposed to sentences implying “fixity” subsequently attributed their poor performance to a seemingly stable and uncontrollable factor (ability) far more than to a factor generally considered to be malleable (effort). Other studies have similarly shown that brief written messages implying a particular theory can activate a “fixed” (or “incremental”) mindset (e.g. Cury et al., 2006; Miele & Molden, 2010).

According to critics, the term “weakness” implies that a characteristic is “fixed” (e.g. Prototype Training Systems, n.d.). Thus, written messages in which “weakness” is used may activate a “fixed” mindset. Whilst in this mindset, individuals may think of a problem as stable and uncontrollable, which may then lower Improvement expectancy (IE). On the other hand, written messages in which “area for improvement” is repeatedly used may activate an *incremental* mindset. Whilst in this mindset, individuals may think of a problem as temporary and controllable. This may then increase IE.

Although no published studies have examined whether “weakness” and “area for improvement” activate different mindsets, some research is suggestive. For example, Hu et al. (2016) conducted an experiment (Study 2) in which participants were given negative feedback about performance on a test. Participants in an “improving belief” condition were then presentd with a few sentences designed to activate the belief that their ability could be improved, for example “you still have a lot of opportunity to improve your reasoning ability” (Hu et al., 2016, p. 79). Participants in this condition reported less negative self-relevant emotion than participants in a control condition. Repeated use of the expression “area for improvement” may have a similar effect. In other words, it may also activate the belief that ability can be improved and have an equally positive psychological impact.

**Figure 1.**
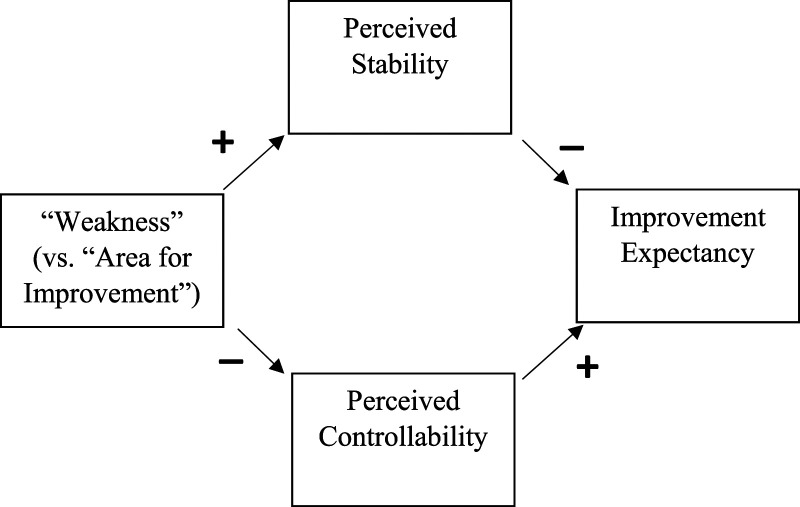
Conceptual mediation model in which “Weakness” (vs. “Area for Improvement”) has a negative indirect effect on improvement expectancy by increasing perceived stability and/or lowering perceived controllability.

In summary, (compared to “area for improvement”) “weakness may enhance perceived stability (PS) and/or lower perceived controllability (PC) by activating a “fixed” (vs. “incremental”) mindset. Greater PS and/or lower PC should then lower improvement expectancy (IE). The hypothesis is depicted in Figure 1.

As noted, expectancy is widely considered to be one of the two main determinants of motivation. The other is *perceived value* (e.g. Wigfield & Eccles, 2000). The possible effects of “weakness” on perceived value of improvement (PVOI) must therefore be considered.

## “Weakness” and Perceived Value of Improvement

If (frequent) use of the term “weakness” lowers improvement expectancy (IE), it may also lower perceived value of improvement (PVOI). However, somewhat paradoxically, “weakness” may also increase PVOI by reducing perceived controllability (PC). Both of these possibilities are explained below.

### Does “Weakness” Reduce Perceived Value of Improvement by Reducing Improvement Expectancy?

Some research suggests that reduced expectancy leads to a decline in perceived value. For example across five different studies, people whose expectations were experimentally lowered (e.g. through negative feedback) devalued future success (Sjåstad et al., 2020). The researchers referred to this phenomenon as the “sour grapes effect.” Other studies have also found that individuals may devalue an outcome if they do not expect to attain it (e.g. Wilson et al., 2004). It has already been suggested that “weakness” might lower improvement expectancy (by increasing perceived stability or lowering perceived controllability). If so, “weakness” might also lower perceived value of improvement - a “sour grapes effect.”

### Does “Weakness” *Increase* Perceived Value of Improvement by Reducing Perceived Controllability?

Popular sources suggest that “weakness” reduces the perceived controllability (PC) of a problem's cause, making the problem seem more difficult to manage (e.g. Rael, 2012). Ironically, reduced PC (and greater perceived difficulty) may actually increase perceived value of improvement). Greater perceived difficulty signals the need for more effort and “during goal pursuit, effort is associated with value” (Labroo & Kim, 2009, p. 128). Many studies suggest that individuals attach more value to goals that are difficult to achieve than to goals that are easy to attain (e.g. Bayuk, 2015; Brehm et al., 1983; Wicker et al., 2005). Wicker et al. (2005) point out that difficult goals signal greater competence if attained (and less incompetence if not) than goals that are easy to achieve. A goal i.e. made to seem difficult to attain may therefore have greater perceived value. Compared to “area for improvement,” therefore, “weakness” may actually *raise* PVOI by lowering PC.

There is however one important qualification. If goal attainment expectancy is overly reduced, then the attractiveness of the goal is reduced too (e.g. Brehm et al., 1983). Moreover, as suggested above, if “weakness” lowers improvement expectancy, it may also indirectly lower perceived value of improvement - a “sour grapes” effect. A positive effect of “weakness” on PVOI should therefore be observed *(only) when improvement expectancy is held constant*. This may be dubbed the “what’s hard is valuable” effect.

**Figure 2.**
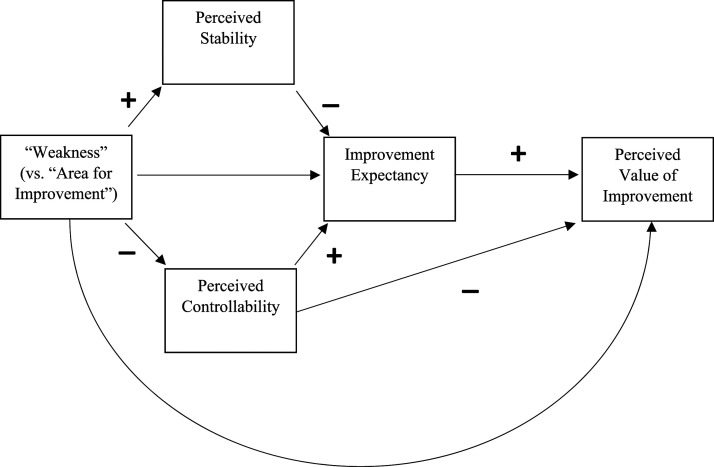
A conceptual mediation model indicating possible effects of “weakness” on Improvement Expectancy (IE) and Perceived Value of Improvement (PVOI) through Perceived Stability (PS) and Perceived Controllability (PC).

It has been suggested so far that “weakness” may lower improvement expectancy (IE) by increasing perceived stability (PS) and/or reducing perceived controllability (PC). It has also been suggested that “weakness” may lower perceived value of improvement (PVOI) by increasing perceived stability (PS) and/or lowering perceived controllability (PC), which lowers IE (which in turn lowers PVOI). However, “weakness” may also *raise* PVOI by lowering PC when IE is held constant - “what’s hard is valuable.” A conceptual model clarifying the basis of the above hypotheses is presented in Figure 2.

Although Figure 2 captures the reasoning so far, it is likely to be incomplete. The effects of “weakness” on the other variables (e.g. perceived stability and controllability) are likely to be moderated by individual differences. Two key individual differences are now introduced.

## Moderating Effects of Perceived Self-Efficacy and Gender (?)

The two possible moderators considered in the present study were (i) perceived self-efficacy and (ii) gender. Reasons for thinking that the effects of “weakness” depend on these variables are presented below.

### Perceived Self-Efficacy as a Potential Moderator

Research indicates that individuals low in perceived self-efficacy (PSE) are more likely to attribute poor performance to stable/uncontrollable causes than individuals high in PSE (Gist & Mitchell, 1992; Silver et al., 1995). Low PSE has also been associated with “fixed” views of the self (e.g. Komarraju & Nadler, 2013). If those low in PSE are more likely to have a “fixed” mindset, they may also be more likely to interpret a “weakness” as stable and uncontrollable than those with high PSE.

Research also suggests that there is a negativity bias in those low in PSE. Individuals with low perceived self-competence are quicker to identify words suggesting failure (e.g. “weak”) than those considering themselves more competent (Tafarodi & Milne, 2002). Using a stroop task, Karademas et al. (2007) found that individuals low in PSE took longer to name the colour of words indicating threat (e.g. “disability”) than they did to name the colour of positive words (e.g. “ableness”). Moreover, they took considerably longer to name the colour of threat-indicating words than individuals with high PSE. Indeed, those with high PSE hardly reacted to threat-indicating words. The authors speculated that those low in PSE may be drawn by such words because they have doubts about their ability to handle threats. On the other hand, those high in PSE pay little attention to words suggesting threats because they are confident in their ability to handle them. This asymmetry may be important for the present study. On the one hand, “weakness” may draw the attention of individuals low in PSE and exert negative psychological effects. On the other hand, those high in PSE may pay less attention to the term. If so, then PSE may moderate the effects of “weakness” on perceived stability and perceived controllability.

**Figure 3.**
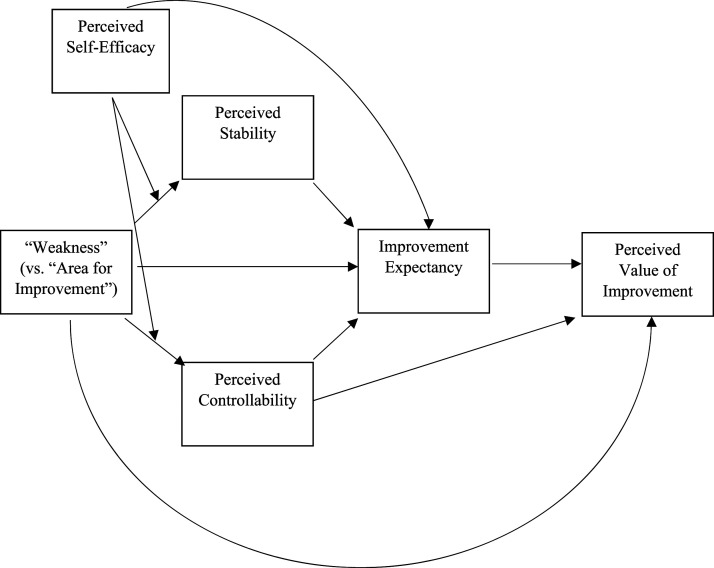
A conceptual model in which perceived self-efficacy moderates the effects of “weakness” on perceived stability and perceived controllability.

PSE is generally considered to be distinct from (outcome) expectancy (e.g. Bandura, 1997). However, individuals with higher perceived self-efficacy in a given domain are likely to have higher improvement expectancy in that domain. PSE should therefore also be directly and positively related to IE. A conceptual model including PSE is presented in Figure 3.

### Gender as a Potential Moderator

Gender differences in language processing are well-documented in the neuroscientific literature (Hill et al., 2006). Males and females are known to differ in their responses to negatively-valenced terms. For example, in a study conducted by Daches Cohen et al. (2003, 2021), negatively-valenced words (e.g. “failure,” “error,” “mistake”) were considered to be considerably more threatening by females than males. Using the Deese-Roediger-McDermott paradigm, Dewhurst et al. (2012) asked men and women to study lists of negatively-valenced and neutral words. Negatively valenced words (e.g. “risk,” “warning,” “trouble” “threat”) were associates of a non-presented lure (e.g. “danger”). Results indicated that females falsely “recalled” more negative lures than males. Females also falsely “recalled” more negative than neutral lures. In addition, the negative lures were rated as more arousing (and the negative study words as more negative) by females than by males. The authors concluded that females reflect on the associations of negative words to a greater extent than males. If so, then they may be more likely to reflect on the negative connotations of “weakness,” e.g. uncontrollability. It may also be relevant that females have lower perceived control than males in various domains (e.g. Simoni et al., 1991). For both of these reasons, “weakness” may be more likely to lower perceived controllability in females, assuming that they are low in PSE. This possibility was investigated in Experiment 2.

It is not clear what gender differences to expect in perceived *internality* (after repeated use of “weakness”). On the one hand, some research suggests that females are more likely than males to make internal attributions for failure (see, e.g., Basow & Medcalf, 1988). On the other hand, there are some reasons for thinking that *males* may be more likely to perceive a “weakness” as internal. Sweeney et al. (1982, p. 361) suggested that “[m]en may be socialized to acknowledge personal responsibility for all of their outcomes, whether they succeed or fail.” Results from their own study indicated that females tended to make external attributions for unsuccessful performance, whereas males tended to make *internal* attributions. Interestingly, males were least distressed when they made internal attributions. The authors speculated that “men may find it less disturbing to accept personal responsibility than to admit that they have lost control over their outcomes” (p. 372). Thus “weakness” may increase perceived internality in males but not females (assuming, again, that they are low in PSE). This possibility was also examined in Experiment 2.

## Recapitulation of Hypotheses

It may be helpful at this point to recapitulate the hypotheses for the present study. According to critics, “weakness” implies stability and uncontrollability, unlike “area for improvement” (e.g. Interview Skills Workbook, nd). It may be the case that (compared to “area for improvement”) “weakness” increases perceived stability and lowers perceived controllability by activating a “fixed” (vs. “incremental”) mindset. However, these effects may be more likely in those with low perceived self-efficacy”. Individuals low in PSE are prone to attribute poor performance to stable/uncontrollable causes (e.g. Silver et al., 1995), are more likely to have a “fixed” (vs. “incremental”) mindset (Komarraju & Nadler, 2013) and are apparently more sensitive to (the connotations of) negative words (e.g. Karademas et al., 2007). In addition, the relative effects of the terms may depend on gender. Females, for example, apparently reflect on the associations of negative words more than males (Dewhurst et al., 2012). All of these considerations suggest the following hypothesis:

H1 In individuals with low perceived self-efficacy (especially females), “weakness” increases perceived stability and/ or lowers perceived controllabilty (compared to “area for improvement”).

Use of the term “weakness” is also thought to lower IE (e.g. Life at United World, 2013). Higher perceived stability and lower perceived controllability have indeed been associated with lower expectancy (Kovenklioglu & Greenhaus, 1978; McMahan, 1973; Rudisill, 1988). Amongst (females) low in perceived self-efficacy (PSE), “weakness” may therefore have a negative indirect effect on improvement expectancy via perceived stability and/or controllability. Formally:

H2 In individuals with low perceived self-efficacy (especially females), “weakness” lowers improvement expectancy by increasing perceived stability and/or lowering perceived controllability.

Research suggests that lower expectancy may lead to lower perceived value – a “sour grapes” effect (Sjåstad et al., 2020; Wilson et al., 2004). If “weakness” lowers improvement expectancy (via perceived stability/controllability), it may then also lower perceived value of improvement. Formally:

H3 In individuals with low perceived self-efficacy(especially females), “weakness” lowers perceived value of improvement by lowering improvement expectancy (by increasing perceived stability and/or lowering perceived controllability).

It was also suggested above that “weakness” may have a *positive* effect on PVOI when expectancy is held constant. More specifically, if (amongst females low in PSE) “weakness” reduces PC (making the issue appear more difficult to manage), then PVOI may increase – “what’s hard is valuable.” In many studies, increased difficulty is indeed positively associated with value (e.g. Wicker et al., 2005). The following hypothesis was therefore formulated:

H4 In individuals with low perceived self-efficacy (especially females), “weakness” has a positive effect on perceived value of improvement (when expectancy is held constant) by lowering perceived controllability.

When individuals are repeatedly exposed to the term “weakness,” there may also be a gender difference in perceived internality. However, the direction of this difference is unclear. On the one hand, some research suggests that females are more likely than males to make internal attributions for failure (see Basow & Medcalf, 1988). On the other hand, males may be more inclined to consider themselves responsible for outcomes (e.g. Sweeney et al., 1982). No firm hypothesis was formulated but Experiment 2 explored whether “weakness” is more likely to enhance perceived internality in males than in females.

## The Present Study

The study consisted of two experiments. Both experiments examined whether perceived self-efficacy moderates the relative effects of “weakness” and “area for improvement” on the dependent variables. Experiment 1 focused on two potential mediators - perceived stability and perceived controllability. In Experiment 2, the focus was expanded to include three additional variables - perceived internality, positive affect and negative affect. In addition, Experiment 2 examined whether effects of the terms – moderated by PSE – are then also moderated by gender.

### Data Availability Statement

Data may be accessed at: https://osf.io/cx492/?view_only=7930dd460a8c467e91e6511804f84f29

## Experiment 1

Experiment 1 was conducted in an all-female secondary school in London. One hundred and one participants were initially recruited (all students in the chosen year group). The sample was not expected to be large enough to test all hypothesised effects. The primary focus of Experiment 1 was on the hypothesised interaction between perceived self-efficacy (a continuous variable) and “weakness/area for improvement” (a categorical variable) in determining perceived stability/controllability. In other words, the main aim was to investigate whether “weakness” (compared to “area for improvement”) increases perceived stability and/or lowers perceived controllability in those with low perceived self-efficacy. If continuous variables have high reliability, then approximately one hundred participants may be sufficient to achieve .8 power to detect an interaction effect of moderate size (Cohen et al., 2003). Much less work has focused on power to detect interactions involving a categorical and a continuous variable (Shieh, 2019). However, such cases have one advantage: the categorical variable (i.e. experimental condition) can be measured with perfect reliability, which increases statistical power considerably.

### Participants

Participants were students preparing for college/university. All students were aged 17–18. On the day of the study, six students opted out and 1 student was ill, leaving a total of 94 participants. There were 45 participants in the “weakness” and 49 in the “area for improvement” condition. Participants had completed a measure of perceived self-efficacy for self-regulation (PSESR) several months prior to the experiment. Ethical approval was provided by the Ethics Committee at Robert Gordon University.

### Procedure

Participants were asked to complete an online survey about self-regulation. In order to clarify the meaning of “self-regulation,” surveys began with a list of examples (e.g. “planning work”). In order to simulate a typical performance review, participants were asked to consider areas in which they were successful (i.e. “strengths”) and areas in which they were *not* successful (i.e. “weaknesses”/“areas for improvement”). Participants in both conditions were initially asked to write down one aspect of self-regulation in which they were successful (e.g. “planning work”). Then they were asked to estimate the impact that this had on their overall performance by selecting from “not much,” “some” or “a lot.” Participants were then asked to write down one aspect of self-regulation in which they were not successful and to estimate its impact in the same manner. After participants had identified the area in which they were *not* successful, this was subsequently referred to as a “weakness” or “area for improvement” depending on condition. The relevant expression was used six times in total in the remainder of the survey. Finally, participants were presented with the items measuring the dependent variables. They were asked to complete these items only for their “weakness/area for improvement.”

### Measures

#### Perceived Self-Efficacy for Self-Regulation (PSESR)

This was assessed by means of the seven-item measure presented by Usher and Pajares (2008). Participants rated their confidence in their ability to do each thing (e.g. concentrate on work) on a 0%–100% scale (α = 0.80). Student scores on this measure have been found to correlate with motivational and achievement variables (Usher & Pajares, 2008).

#### Improvement Expectancy (IE)

This was assessed by the three-item measure used by Abdulla and Woods (2021a, 2021b, 2021c, 2021d). Participants were asked to provide answers on a 0–10 scale for each item (e.g. “How likely is it that you will improve in this area?”). Higher scores indicated higher IE (α = 0.67). Scores on this measure have been found to correlate strongly with scores on measures of commitment (e.g. Abdulla & Woods, 2021a).

#### Perceived Value of Improvement (PVOI)

This was measured by three items derived from Huang et al. (2017). Participants were asked to provide answers on a 0–10 scale for each item (e.g. “How much value do you see in improving in this area?”). Higher scores indicated higher PVOI (α = 0.91). Huang et al. (2017) report the expected correlation between scores on this measure and scores on a measure of motivation.

#### Perceived Controllability (PC)

This was measured by the three “personal control” items on the Revised Causal Dimension Scale (McAuley et al., 1992). To facilitate comprehension, items were phrased as questions and participants were asked to provide answers on a 1–9 scale for each item (e.g. “To what extent is it something over which you have power?”). Higher scores indicated higher PC (α = 0.76). McAuley et al. (1992) report evidence of the factorial validity for the items.

#### Perceived Stability (PS)

This was measured by the three “stability” items on the Revised Causal Dimension 13 Scale (McAuley et al., 1992). Answers were provided on a 1–9 scale for each item (e.g. “To what extent is it stable over time?”). McAuley et al. (1992) present evidence of factorial validity for the items. Higher scores indicated higher PS (α = 0.64).

### Analytical Strategy

Hypothesised effects in the present study were conditional effects. That is, the effects of “weakness” (vs. “area for improvement”) were hypothesised to depend on participants' perceived self-efficacy (PSE). Nevertheless, one-way ANCOVAs (with PSE as the covariate) were also performed in order to determine whether there were any statistically significant *overall* group differences on the dependent variables. Moderated multiple regression was then used to investigate whether the effect of “weakness” on perceived stability/controllability depends on perceived self-efficacy for self-regulation. If evidence for moderation was found, the Johnson-Neyman (JN) procedure was used to identify regions of statistical significance (Hayes, 2018). 95% bootstrapped confidence intervals (CIs) for indirect effects were based on 5000 bootstrapped samples.

The inclusion of multiple parallel correlated mediators (e.g. perceived stability, controllability, internality) has one important drawback - it reduces the power of tests for specific indirect effects (Hayes, 2018). In the present study, this problem was addressed as follows. In the initial analysis both (or all) parallel mediators were included. If a “mediator” was not significantly affected by condition (at least at some level of PSESR) or not significantly related to improvement expectancy, then that “mediator” was dropped. After this “Model Pruning,” the analysis was reconducted. This approach has two advantages. First, removing variables that apparently have little or no effect leads to a more parsimonious model (e.g. Maxwell et al., 2017). Second, when there are fewer correlated predictor variables in the model (estimated) standard errors tend to be reduced, which increases statistical power to detect specific indirect effects (Hayes, 2018). Nevertheless, both sets of analyses (initial and “pruned”) are presented so that readers may compare results. Whenever the words “significant,” “significance” and “significantly” are used, they should be understood in the statistical sense (i.e. *p* < .05).

### Results

All participants provided responses to all questions. Table 1 presents descriptive statistics for the two conditions.

**Table 1.** Means and Standard Deviations for the Two Conditions.

	“Weakness”		“Area for Improvement”	
	M	SD	M	SD
PSESR	68.80	9.37	67.04	12.48
IE	5.97	1.15	5.66	1.02
PVOI	7.82	1.70	7.70	1.61
PC	6.14	1.14	5.62	1.42
PS	4.13	1.26	4.20	1.09

*Note.* PSESR, Perceived Self-Efficacy for Self-Regulation; IE, Improvement Expectancy; PVOI, Perceived Value of Improvement; PC, Perceived Controllability; PS, Perceived Stability.

One-way ANCOVAs for each of the dependent variables (IE, PVOI, PC and PS) with PSE as a covariate yielded no significant overall differences between conditions.

#### Interactions Between “Weakness” (vs. “Area for Improvement”) and Perceived Self-Efficacy for Self-Regulation

In the model of perceived controllability, the interaction between condition (“Weakness” vs. “Area for improvement) and PSESR was not significant: *b* = .01[−.04, .06], *t* = .42, *p* = .68. In the model of perceived stability, however, the interaction between condition and PSESR was significant: *b* = −.05 [−.09, −.00], *t* = 2.13, *p* = .04. At lower levels of PSESR, “weakness” apparently increased PS. The Johnson-Neyman (JN) technique indicated that this effect was right at the cut-off for significance (*p* = .05) at PSESR levels ≤43%. Two participants had PSESR scores in this range. At the lowest level of PSESR observed in the data (40%) “weakness” was estimated to increase the perceived stability of the problem by 1.31[.00, 2.63] points - a considerable effect.

**Figure 4.**
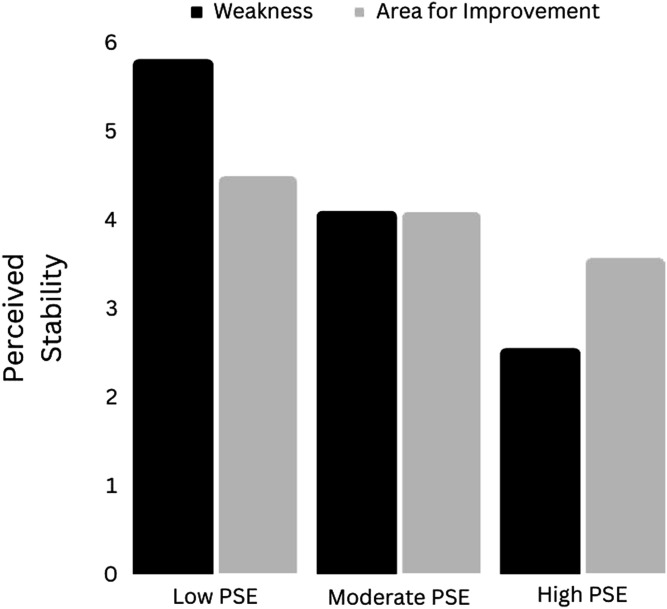
The effect of “Weakness” (vs. “Area for Improvement”) on perceived stability at different levels of perceived self-efficacy for self-regulation. *Note.* PSE = Perceived Self-Efficacy.

At levels of PSESR ≤43%, “weakness” was associated with a significant reduction in perceived stability. However, only one participant had a PSESR score in this range. At this level of PSESR, “weakness” was associated with a PS score 1.32[−2.62, −.02] points lower than “area for improvement” - another considerable difference. Figure 4 depicts the moderation effect. At very low levels of PSESR, “weakness” apparently increased the perceived stability of the problem. At extremely high levels of PSESR, on the other hand, perceived stability was estimated to be lower in the “weakness” condition. At a moderate level of PSESR - 67.88% (the sample mean) - perceived stability means were essentially identical in the two conditions

.

#### Indirect Effect of “Weakness” on Improvement Expectancy Through Perceived Stability

The index of moderated mediation (.01) was associated with a CI excluding zero [.0014, .0266]. There was therefore evidence to suggest that the indirect effect of “weakness” on improvement expectancy (through perceived stability) was moderated by perceived self-efficacy for self-regulation (PSESR). When PSESR was very low (40%–46%), “weakness” was estimated to lower improvement expectancy by increasing perceived stability. At the lowest observed level of PSESR (40%), this effect was estimated to be −.33[−.7804, −.0118], which was almost one-third of a standard deviation (0.29) - a small but non-negligible effect. At very high levels of PSESR, “weakness” was estimated to have a positive indirect effect on IE. At the highest observed level of PSESR (96%), this effect was estimated to be +.33[.0381, .7460] - again, a small but potentially meaningful effect.

### Other Results

All other estimated effects on IE and PVOI were not statistically different from zero.

### Model Pruning

Perceived controllability was not significantly affected by condition. Furthermore, the indirect effect on improvement expectancy through perceived controllability was not statistically different from zero. Perceived controllability was therefore dropped from the model and the analysis was conducted with perceived stability alone. The only important difference in the results was that the estimated indirect effects of “weakness“ on improvement expectancy through perceived stability were now somewhat larger. The indirect effect was estimated to be −.51[−1.0808, −.0353] at the lowest level of PSESR (40%), and .52[.0850, 1.0295] at the highest level (96%). These amount to almost a half of a standard deviation - moderately large effects.

## Brief Discussion

Experiment 1 suggested that (compared to “area for improvement”) use of the term “weakness” may indeed increase perceived stability, at least amongst those with very low perceived self-efficacy for self-regulation. In addition, results suggested that this increased perceived stability reduces improvement expectancy. At extremely high levels of perceived self-efficacy for self-regulation, “weakness” was actually estimated to enhance improvement expectancy by reducing perceived stability. Results of Experiment 1 therefore support the hypotheses that (compared to “area for improvement”) “weakness” increases perceived stability in females with low perceived self-efficacy (H1) and that “weakness” has a negative indirect effect on improvement expectancy by increasing perceived stability (H2).

However, limitations of the experiment must be acknowledged. First, the size of the sample meant that only some of the hypothesised effects could be adequately tested. The lack of evidence for the expected interaction between condition and perceived self-efficacy in predicting perceived controllability may have been due to relatively low power. Second, all participants in Experiment 1 were female. As noted in the introduction, it seems possible that the effects of the terms differ across genders. Third, all participants in Experiment 1 were of the same age and attended the same school and may not be representative of “females” as a whole. These limitations were addressed in Experiment 2.

## Experiment 2

The much larger sample in Experiment 2 allowed three additional potential mediators to be examined: perceived internality, positive affect and negative affect. These variables have been theoretically (and empirically) linked to expectancy and perceived value of improvement. For example, individuals primed with negative words experience more negative affect than individuals primed with positive words (Chartrand et al., 2006). Negative affect leads to reductions in hope (Jordens & Van Overwalle, 2005) and (at least in females) optimism (Lewis et al., 1995). On the other hand, positive affect induced by positive words leads to higher expectancy (Erez & Isen, 2002). “Weakness” [“Area for improvement”] may then have negative [positive] effects on expectancy by eliciting negative [positive] affect.

### Does “Weakness” Increase Perceived Value of Improvement by Increasing Perceived Internality?

If “weakness” leads to greater perceived internality, this may have an effect on perceived value of improvement (PVOI). Myers et al. (2014, p. 4) refer to research “that finds *internal attribution* a necessary condition for motivating learning and behavior change” (italics added). The results of their own experiments suggest that internal attributions for failure lead to greater efforts to improve. In addition, when individuals consider themselves responsible for negative outcomes, they often increase personal investment (Staw, 1976). According to critics, informing individuals that they have a “weakness” implies that there is some sort of issue *within them* (e.g. Connolly & White, 2017). If perceived internality is thereby enhanced, this may then increase the PVOI. However, this effect may appear only in those with low PSE. As noted in the introduction, individuals with low PSE are especially sensitive to negative words, whereas those high in PSE may ignore them (e.g. Karademas et al., 2007).

### Do the Effects of “Weakness” Also Depend on Gender?

As explained in the introduction, there are reasons for thinking that the effects of “weakness” on the dependent variables (e.g. perceived controllability and internality) depend on gender as well as PSE. For example, many studies indicate that females have lower perceived control (over various problems) than males (e.g. Dickens & Perry, 1982; Lewis et al., 1999; Simoni et al., 1991). Perceived controllability may therefore be more likely to drop at the insinuation of a “weakness” in females than in males, especially if those females are also low in PSE.

On the other hand, males may be more likely than females to make “internal” attributions (Sweeney et al., 1982). “Weakness” may then increase perceived internality in males (but not in females), assuming, again, that those males are low in PSE.

One of the aims of Experiment 2 was therefore to test for moderated moderation (i.e. three-way interactions). For example, if “weakness” increases perceived internality at low (but not high) levels of PSE, perhaps this effect is greater in males than females. Conversely, if “weakness” lowers perceived controllability at low (but not high) levels of PSE, perhaps this effect is greater in females than in males.

### Participants

Three hundred and forty-three individuals were recruited through Prolific and randomly assigned to the “weakness” or “area for improvement” condition. Participants on Prolific are more honest, diverse and experimentally naive than those on Amazon Mechanical Turk (Peer et al., 2017). Of those assigned to the “weakness” condition, 93% (160 out of 172) completed the study. Of those assigned to the “area for improvement” condition 95% (162 out of 171) completed the study. Those who did not complete the study did not supply any demographic or experimental data. Participants were English-speaking undergraduate or postgraduate students aged between 18 and 35 (M = 21.42; SD = 3.47). One hundred and fifty-three described themselves as male (47.5%); 162 as female (50.3%) and 7 as “other” (2.2%). Thirty-six percent were from the US (116 participants); 26% were from the UK (85 participants); 7.5% were from Canada (24 participants); 6.5% were from South Africa; and the remainder reported various other nationalities including Australian, Irish and Malaysian.

### Procedure

The procedure was identical to that in Experiment 1 except for the following differences. Experiment 1 was conducted in a school and each participant wrote answers on a sheet of paper. Experiment 2 was conducted through the internet. The online materials were divided into several pages, each of which had a heading. In Experiment 1, each term (i.e. “weakness”/“area for improvement”) was used six times in total. In Experiment 2, each term was used 17 times in total. This increase was achieved as follows. First, in the online version of the intervention (used in Experiment 2), the relevant term was incorporated into the heading of all relevant pages. Second, the relevant term was included in the pages devoted to the dependent measures. It seemed possible that increasing the frequency of the term(s) would increase the size of the effects. Study materials may be obtained from the corresponding author.

### Measures

#### Perceived Self-Efficacy for Self-Regulation (PSESR)

This was measured using the same instrument as in Experiment 1 (α = .82).

#### Improvement Expectancy (IE)

Online pilot studies indicated that the estimated reliability of the IE measure was considerably enhanced by including an additional item: “How confident are you that you could improve in this area (if you wanted to)?” Confirmatory factor analysis supported the assumption of unidimensionality for the resulting four-item measure, which was therefore used. Estimated reliability was indeed improved (α = .81)

#### Perceived Value of Improvement (PVOI)

This was measured using the same instrument as in Experiment 1 (α = .90)

#### Perceived Controllability (PC)

This was measured using the same instrument as in Experiment 1 (α = .82)

#### Perceived Stability (PS)

This was measured using the same instrument as in Experiment 1 (α = .62)

#### Perceived Internality (PI)

This was measured by the three “locus of causality” items on the Revised Causal Dimension Scale (McAuley et al., 1992). Answers were provided on a 1–9 scale. Higher scores indicated higher perceived internality (α = 0.66). McAuley et al. (1992) provide evidence of factorial validity.

#### Positive Affect (PA) and Negative Affect (NA)

These were measured by means of the Positive and Negative Affect Schedule - PANAS (Watson et al., 1988). Reliability estimates were very high for both positive affect (α = 0.91) and negative affect (α = 0.89). Participants were asked to rate how they felt “right now.”

### Results

All participants responded to all questions. Table 2 presents descriptive statistics for the two conditions.

**Table 2.** Means and Standard Deviations for the Two Conditions.

	“Weakness”		“Area for Improvement”	
	M	SD	M	SD
PSESR	63.85	14.49	65.05	14.78
IE	5.43	1.49	5.53	1.49
PVOI	6.99	1.88	6.95	1.88
PC	5.67	1.44	6.14	1.47
PS	4.63	1.35	4.54	1.21
PI	6.86	1.30	6.79	1.39
PA	24.48	8.72	25.39	8.37
NA	24.87	9.14	23.92	9.02

*Note.* PSESR, Perceived Self-Efficacy for Self-Regulation; IE, Improvement Expectancy; PVOI, Perceived Value of Improvement; PC, Perceived Controllability; PS, Perceived Stability; PI, Perceived Internality; PA, Positive Affect; NA, Negative Affect.

One-way ANCOVAs with perceived self-efficacy (PSESR) as the covariate yielded one significant overall between-condition difference. With PSESR controlled, “weakness” apparently lowered perceived controllability (compared to “area for improvement”) by almost half a point: *b* = −.41 [−.70, −.12], *t* = 2.80, *p* = .006.

#### Conditional Effects of “Weakness” on Perceived Controllability

In the model of perceived controllability, the three-way interaction between gender, condition and perceived self-efficacy for self-regulation (PSESR) was significant: *F(*1,307) = 6.23, *p* = .01, Δ*R*^2^ = .02. Probing of this interaction revealed that PSESR was a significant moderator of the effect of “weakness” on perceived controllability amongst women (*p* = .03), but not amongst men (*p* = .19). The “Weakness” condition was estimated to lower perceived controllability for both genders, but for women this negative effect was more pronounced at lower levels of perceived self-efficacy for self-regulation (PSESR).

**Figure 5.**
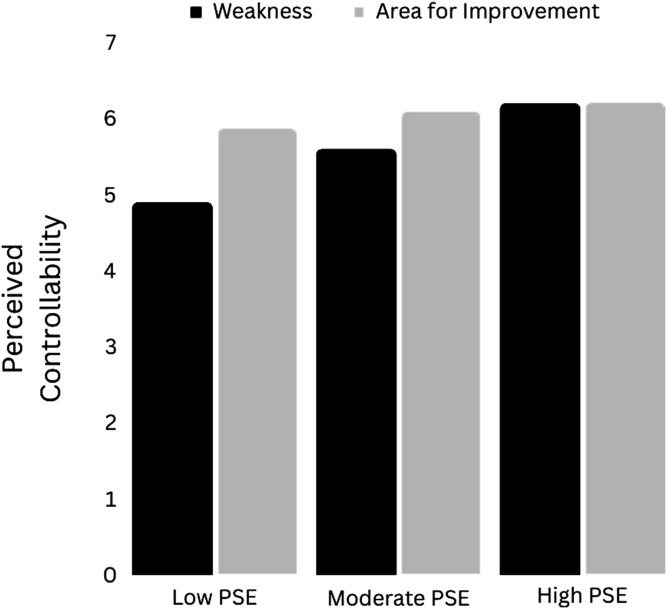
The effect of “Weakness” (vs. “Area for Improvement”) on perceived controllability in women at low, moderate and high levels of perceived self-efficacy for self-regulation. *Note.* PSE = Perceived Self-Efficacy.

Figure 5 depicts the effect of “weakness” (vs. “area for improvement”) on perceived controllability in women at a low (mean 1 SD), moderate (mean) and high (mean +1 SD) level of PSESR. When PSESR was low (49.93%), “weakness” was estimated to lower perceived controllability (relative to “area for improvement”) by almost one point - an appreciable effect: b = .98 [1.59, .36], t = 3.12, p = .002. When PSESR was at the sample mean (64.54%), “weakness” was estimated to lower perceived controllability in women by approximately half a point: b = .50 [.90, .10], t = 2.46, p = .01. When PSESR was high (79.15%), perceived controllability was estimated to be all but identical in the two conditions: b = .02 [.58, 54], t = .07, p = .94.

#### Conditional Effects of “Weakness” (vs. “Area for Improvement”) on Perceived Internality

**Figure 6.**
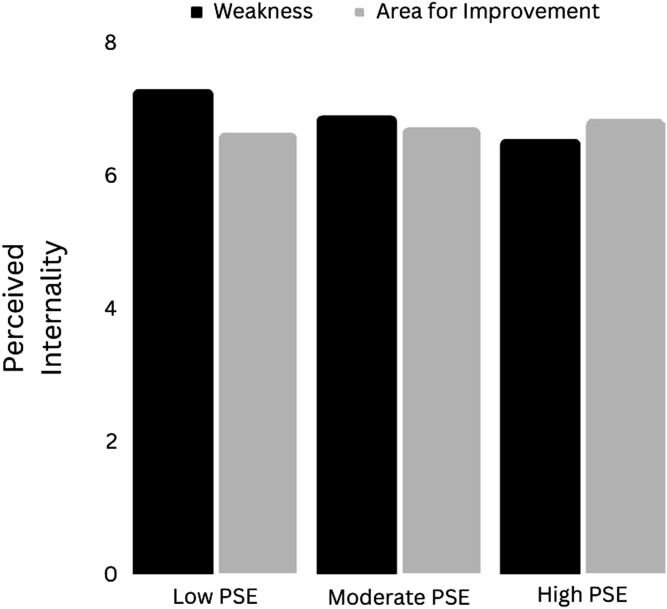
The effect of “Weakness” versus “Area for Improvement” on perceived internality in men at low, moderate and high levels of perceived self-efficacy for self-regulation. *Note.* PSE = Perceived Self-Efficacy.

In the model of perceived internality, the three-way interaction between gender, condition and PSESR was significant: *F*(1,307) = 7.24, *p* = .01, Δ*R*^2^ = .02. Probing of this interaction indicated that PSESR was a significant moderator of the effect of “weakness” on perceived internality amongst men (*p* = .01), but not amongst women (*p* = .14). Figure 6 depicts the effect of “weakness” on perceived internality in men at low (mean −1 SD), moderate (mean) and high (mean +1 SD) levels of PSESR. At a low level of PSESR (49.93%), “weakness” was estimated to increase perceived internality by over half a point - a moderately large effect: *b* = .64[.08, .120], *t* = 2.25, *p* = .02. At a moderate level of PSESR (64.54%), “weakness” was associated with a small, non-significant increase in perceived internality: *b* = .16[−.26, .58], *t* = .74, *p* = .46. At a high level of PSESR (79.15%), “weakness” was associated with lower perceived internality but the difference was not significant: *b* = −.32 [−.92, .28], *t* = 1.06, *p* = .29.

#### Conditional Indirect Effects of “Weakness” on Improvement Expectancy Through Perceived Controllability

For the indirect effect of “weakness” on improvement expectancy (IE) through perceived controllability (PC), the CI was extremely close to excluding zero [.0001, .0455]. The CI for conditional moderated mediation included zero for men [−.0233, .0075] but was extremely close to excluding zero for women [−.0009, .0303]. “Weakness” was estimated to lower IE by lowering PC for both genders at low, moderate and high levels of PSE. CIs for these effects excluded zero except for men with low PSE and women with high PSE. Amongst women, when PSESR was low (49.93%), “weakness” was estimated to lower IE via reduced PC by −.43 [−.81, −.08], a small but non-negligible effect. At the lowest observed level of PSESR (17.14%), this effect was estimated to be almost one point on the 0–10 scale: −.91[−1.77, −.01], an appreciable effect.

#### Indirect Effects of “Weakness” on Perceived Value of Improvement Through Perceived Internality

For the indirect effect of “weakness” through perceived internality alone, the index of moderated moderated mediation (.02) was associated with a CI excluding zero [.0035, .0373]. The index of conditional moderated mediation for women (.01) was associated with a CI including zero [−.0026, .0194]. The index of conditional moderated mediation for men (−.01), however, was associated with a CI excluding zero [−.0238, −.0005].

Amongst men, at a low level of PSESR (49.93%), “weakness” was estimated to increase PVOI (through increased perceived internality) by almost a quarter of a point: .21[.0216, .4676]. At a moderate level of PSESR (64.54%) the increase in PVOI was estimated to be much smaller (.05) and the CI included zero [−.0810, .2158]. At a high level of PSESR (79.15%), the indirect effect of “weakness” on PVOI was estimated to be negative (−.11) but the CI again included zero [−.3357, .1156].

### Other Results

All other estimated effects were not statistically different from zero.

### Model Pruning

Perceived controllability (PC) was the only putative mediator significantly related to both condition and improvement expectancy (IE). All parallel mediators except for PC were therefore removed in order to allow a more powerful test of indirect effects through PC.

With IE as the dependent variable, the index of moderated moderated mediation (.01) was associated with a CI that excluded zero [.0011, .0573]. The index of conditional moderated mediation for men (−.01) clearly included zero [−.0295, .0102]. However, the index for women (.02) was associated with a CI that all but excluded zero [.0000, .0392].

For women, the negative indirect effect of “weakness” on IE through reduced PC was most pronounced when PSESR was low. At one standard deviation below the mean on the PSESR scale (49.93%), “weakness” was estimated to lower IE in women (by reducing PC) by over half a point: −.57[−1.0204, −.1086], a moderately large effect. At the lowest observed value of PSESR (17.14%), the reduction in IE (in women) was estimated to be −1.20[−2.2695, −.1376], a large effect. When PSESR was at the sample mean (64.54%), “weakness” was estimated to reduce IE in women (via reduced PC) by −.29[−.5487, −.0426] - a small effect. At a high level of PSESR (79.15%), the negative effect on IE was estimated to be extremely small (−.01) and was not statistically different from zero [−.3056, .2838].

The indirect effects of “weakness” on PVOI through reduced PC (alone) were estimated to be positive for both genders at low, moderate and high PSESR (“What’s hard is valuable”). CIs for many of these effects excluded zero, as indicated in Table 3. Amongst women with low PSESR, the positive effect of “weakness” on PVOI via reduced PC was estimated to be approximately a quarter of a point on the 0–10 scale - a small effect.

**Table 3.** Estimated Indirect Effects of “Weakness” on Perceived Value of Improvement Through Perceived Controllability at Low, Moderate and High Levels of Perceived Self-Efficacy for Self-Regulation.

Gender	Level of PSESR	Estimated Effect	95% CI
Male	49.93% (“Low”)	.0647	[−.1078, .2930]
	64.54% (“Moderate”)	.1264	[.0042, .3043]
	79.15% (“High”)	.1881	[.0067, .4365]
Female	49.93% (“Low”)	.2386	[.0039, .5555]
	64.54% (“Moderate”)	.1217	[−.0018, .2893]
	79.15% (“High”)	.0048	[−.1388, .1464]
Index of moderated moderated mediation: −.0122 [−.0310, .0009]			
Indices of conditional moderated mediation: Males: .0042 [−.0046, .0154] Females: −.0080 [−.0208, .0007]			

*Note.* PSESR, Perceived Self-Efficacy for Self-Regulation.

In the pruned model, “weakness” was estimated to *reduce* PVOI through PC and IE in sequence. That is, “weakness” apparently lowered perceived controllability, which in turn lowered improvement expectancy, which then lowered perceived value of improvement. This negative (estimated) indirect effect on PVOI was observed for both genders at low, moderate and high PSE (and is consistent with the “sour grapes” hypothesis). Estimated effects are presented in Table 4. As will be observed, CIs for most effects exclude zero. Moreover, as indicated in Table 4, the CI for the index of moderated moderated mediation excluded zero and the CI for the index of conditional moderated mediation in women was right on the verge of excluding zero.

**Table 4.** Estimated Indirect Effects of “Weakness” on Perceived Value of Improvement Through Perceived Controllability and Improvement Expectancy in Sequence at Low, Moderate and High Levels of Perceived Self-Efficacy for Self-Regulation.

Gender	Level of PSESR	Estimated Effect	95% CI
Male	49.93% (“Low”)	−.0591	[−.2296, .0913]
	64.54% (“Moderate”)	−.1155	[−.2430, −.0209]
	79.15% (“High”)	−.1718	[−.3654, −.0322]
Female	49.93% (“Low”)	−.2180	[−.4355, −.0390]
	49.93% (“Low”)	−.1112	[−.2307, −.0146]
	49.93% (“Low”)	−.0044	[−.1174, .1199]
Index of moderated moderated mediation: .0122 [.0005, .0253]			
Indices of conditional moderated mediation: Males: −.0039 [−.0129, .0035] Females: .0073 [−.0001, .0168]			

*Note.* PSESR, Perceived Self-Efficacy for Self-Regulation.

There was therefore evidence to suggest that the indirect effect of “weakness” on perceived value of improvement (PVOI) through perceived controllability and improvement expectancy was moderated by perceived self-efficacy in women. At the lowest observed level of PSESR (17.14%), the negative indirect effect of “weakness” on women’s PVOI was estimated to be −.46[−.9832, −.0450], a small effect.

### Supplementary Analyses

In the analyses above positive and negative affect were tested as mediators of the effects of “weakness” on improvement expectancy. However, positive/negative affect may also be a *consequence* of increased/reduced expectancy (e.g. Sniezek, 1999). Greater perceived internality might also influence affect. For example, if the cause of poor performance is perceived to be internal this might lead to negative emotions such as shame (Weiner, 2012). Supplementary analyses were therefore conducted in which positive and negative affect were modelled as consequences rather than causes of improvement expectancy (see the Supplemental Material).

### Brief Discussion

In Experiment 2, “weakness” apparently had a negative overall effect on perceived controllability (relative to “area for improvement”). However, amongst women, this effect was (as hypothesised) moderated by perceived self-efficacy for self-regulation. That is, the reduction in perceived controllability in women was especially pronounced at low levels of perceived self-efficacy. On the other hand, at high levels of perceived self-efficacy, “weakness” did not appear to reduce perceived controllability. In many aspects of life, females report lower perceived control than males (Dickens & Perry, 1982; Lewis et al., 1999; Simoni et al., 1991). Negative biases in the processing of words have been observed both in females (e.g. Daches Cohen et al., 2003) and more generally in individuals with low PSE (e.g. Tafarodi & Milne, 2002). The combination of “weakness,” female gender *and* low PSE may then make “uncontrollable” inferences especially likely. Furthermore, such inferences may then lead to a reduction in improvement expectancy. This negative indirect effect was again estimated to be greatest amongst women with low perceived self-efficacy.

The estimated effects of “weakness” on perceived value of improvement (PVOI) were (as predicted) both positive and negative and (in the pruned model) significantly different from zero. On the one hand, “weakness” apparently lowered perceived controllability, which then lowered improvement expectancy, which finally lowered perceived value of improvement. This is consistent with the “sour grapes” hypothesis. Once again, this effect was most pronounced in females with low perceived self-efficacy. On the other hand, with improvement expectancy controlled, “weakness” also appeared to raise perceived value of improvement by lowering PC, suggesting a “what's hard is valuable” effect. As before, this effect was most pronounced in females with low perceived self-efficacy.

With regard to perceived internality, it was males (not females) who were seemingly most affected. That is, “weakness” appeared to increase perceived internality but only at low levels of perceived self-efficacy for self-regulation. This increase in perceived internality was then associated with a small increase in perceived value of improvement. In other words, amongst males with low perceived self-efficacy, “weakness” appeared to increase perceived internality, which in turn increased perceived value of improvement. This is consistent with research indicating that internal attributions for negative states of affairs inspire greater efforts to improve (Myers et al., 2014).

Supplementary analyses suggested that “weakness” may also lead to reductions in positive affect, the mechanisms depending on gender (see “Supplemental Material”). For women with low PSE (for self-regulation) results suggested that “weakness” may lead to a small reduction in positive affect by reducing perceived controllability, which then reduces improvement expectancy (which in turn reduces positive affect). For men with low PSE, results suggested that “weakness” may lower positive affect by increasing perceived internality (which in turn reduces positive affect). However, both of these indirect effects of “weakness” on affect were small.

In both Experiment 1 and Experiment 2, “weakness” apparently had a negative indirect effect on improvement expectancy in those with low perceived self-efficacy. However, the mediating variable differed. The mediator appeared to be perceived stability in Experiment 1 and perceived controllability in Experiment 2. The difference may be related to the greater (estimated) reliability of data for perceived controllability in Experiment 2. Alternatively, it may be related to the samples. Experiment 1 involved a sample of (mostly) English females aged 17–18 attending the same independent school in London. Experiment 2 involved male and female undergraduates aged 18–35 from various colleges/universities around the world. Some research suggests that perceived stability and perceived controllability have different psychological effects in students of different cultures/nationalities (e.g. Betancourt & Weiner, 1982). It may also be the case that perceived stability and perceived controllability are affected differently by “weakness” from one culture/nationality to another. Future studies could investigate this possibility.

## General Discussion

It is widely assumed that the term “weakness” leads people to infer that they have a stable, uncontrollable and internal problem (e.g. Boroson, 2011; Rael, 2012; Ripp, 2012; Spiegel, 1994; Spinella, 2020). Results of the present study suggest that these fears are not entirely misplaced. There are, however, important qualifications.

Taken together, Experiments 1 and 2 indicate that “weakness” may indeed increase perceived stability and/or reduce perceived controllability and enhance perceived internality. However, in both experiments such effects were most apparent *when perceived self-efficacy was low*. Individuals low in PSE are more likely to be affected by negative terms than those who are high in PSE (e.g. Karademas et al., 2007). They are also more like to have a “fixed” view of the self (Komarraju & Nadler, 2013) and to make “stable” and “uncontrollable” attributions (Gist & Mitchell, 1992; Silver et al., 1995). In the present study, at moderate and high levels of PSESR “negative” effects of “weakness” were less pronounced or even non-existent. PSE for many individuals is moderate rather than extremely high or low. Thus, for many individuals “weakness” may not have the widely feared “negative” effects (e.g. a reduction in the PC of the problem).

Furthemore, whether the effects of “weakness” on perceived controllability and perceived internality should be described as “negative” is open to question. Although it is true that lower perceived controllability may lower improvement expectancy and consequently reduce perceived value of improvement (“sour grapes”), it also appears that lower perceived controllability (engendered by “weakness”) may enhance perceived value of improvement when improvement expectancy is held constant (“what's hard is valuable”). These negative and positive indirect effects may ultimately cancel each other out.

The other important finding to emerge from the study relates to gender. As noted, it appears that when perceived self-efficacy is relatively low, “weakness” may lead to greater perceived stability, lower perceived controllability or greater perceived internality. However, gender apparently also plays a role. Perceived self-efficacy appeared to moderate the effect of “weakness” on perceived controllability in females but not in males. In addition, amongst those low in PSESR, males (but not females) were apparently more likely to infer internality. Future research should attempt to replicate these findings.

Limitations of the present study should also be considered. Participants in both experiments were allowed to choose whatever aspect of self-regulation they considered problematic. Thus, the focal aspect for one participant might be “planning work” whereas for another it might be “procrastination.” Allowing participants to choose their own focal aspect increases the external and ecological validity of the study. However, it does have one drawback: inter-individual differences in focal aspect add “noise” (i.e. additional extraneous variance), which makes between-group differences more difficult to detect. The relative effects of “weakness” and “area for improvement” may be smaller or greater for one aspect of self-regulation (e.g. planning work) than for another (e.g. procrastination), thus blurring any overall effect of condition. Future studies could address this limitation by requiring all participants to consider the same aspect of self-regulation.

It must also be remembered that participants were presented with the words “weakness” and “area for improvement” in a written/virtual format. The advantage of the written/virtual approach is that it makes it possible to focus on the relative effects of the expressions themselves without human or social “contamination.” However, it should be acknowledged that differences between written/virtual and oral/face-to-face communication may have an impact on the way in which the two expressions are processed. Dual process models of persuasion such as the heuristic-systematic model (Chen & Chaiken, 1999) predict that a message conveyed through text is more likely to be processesed *systematically* whereas a message conveyed orally is more likely to be processed *peripherally* or *heuristically* (e.g. Guadagno & Cialdini, 2007). When processing oral communication peripherally/heuristically, individuals may be influenced by characteristics of the speaker, e.g. attractiveness or perceived likeability (e.g. Chaiken & Eagly, 1983). The relative effects of “weakness” and “area for improvement” may therefore be different in a face-to-face interaction between a teacher and a student (or manager and employee). Nevertheless, it should be noted that *written* self-assessments including the term “weakness” or “area for improvement” are common in education and the work place (e.g. Doyle, 2005; Jankowska & Zielińska, 2015; Lee & Everett, 2004; Violanti & Kelly, 2023).

## Conclusion

The popular literature contends that “weakness” is demoralising and should be replaced by “area for improvement.” The present study is the first to address the matter experimentally. Compared to the alternative “area for improvement,” “weakness” does appear to have some negative effects, especially in those with low perceived self-efficacy. However, further investigation is required. Future research should examine whether the results of the present study can be replicated in this domain (i.e. self-regulation) and other domains (e.g. physical activity, interpersonal communication, use of information technology, etc.). Given the ubiquity of the terms and the fears about “weakness,” the importance of such research should be clear. Ultimately, major terminological recommendations in education and the workplace should be based on data rather than intuition.

## Supplemental Material

Supplemental Material - Should We Really Be Afraid of “Weakness”? Applying the Insights of Attribution Theory Supplemental Material for Should We Really Be Afraid of “Weakness”? Applying the Insights of Attribution Theory by Adam Abdulla in Psychological Reports

## Data Availability

All data generated or analysed during this study are included in this published article.

## References

[bibr1-00332941241231210] Abdulla A. Woods R. (2021a). The effects of current unsatisfactory performance and evaluative approach on improvement expectancy and commitment to improvement. Motivation and Emotion, 45(2), 159–170. 10.1007/s11031-021-09864-8

[bibr2-00332941241231210] Abdulla A. Woods R. (2021b). Obstacles vs. resources - comparing the effects of a problem-focused, solution-focused and combined approach on perceived goal attainability and commitment. International Journal of Applied Positive Psychology, 6(2), 175–194. 10.1007/s41042-020-00044-6

[bibr3-00332941241231210] Abdulla A. Woods R. (2021c). The effects of current unsatisfactory performance and evaluative approach on improvement expectancy and commitment to improvement. Motivation and Emotion, 45(2), 159–170. 10.1007/s11031-021-09864-8

[bibr4-00332941241231210] Abdulla A. Woods R. (2021d). The effect of solution-focused scaling and solution-focused questions on expectancy and commitment. School Psychology Review, 52(6), 709–720. 10.1080/2372966X.2021.1942196

[bibr5-00332941241231210] Adair J. (2004). John Adair’s 100 greatest ideas for effective leadership and management. Capstone.

[bibr6-00332941241231210] Bandura A. (1997). Self-efficacy: The exercise of control. W H Freeman/Times Books/Henry Holt & Co.

[bibr7-00332941241231210] Basow S. A. Medcalf K. L. (1988). Academic achievement and attributions among college students: Effects of gender and sex typing. Sex Roles, 19(9–10), 555–567. 10.1007/bf00289735

[bibr8-00332941241231210] Bayuk J. (2015). Should I plan? Planning effects on perceived effort and motivation in goal pursuit. Journal of Consumer Behaviour, 14(5), 344–352. 10.1002/cb.1525

[bibr9-00332941241231210] Bell B. S. (2017). Strategies for supporting self-regulation during self-directed learning in the workplace. In Ellington J. E. Noe R. A. (Eds.), Autonomous learning in the workplace (pp. 117–134). Routledge.

[bibr10-00332941241231210] Betancourt H. Weiner B. (1982). Attributions for achievement-related events, expectancy, and sentiments: A study of success and failure in Chile and the United States. Journal of Cross-Cultural Psychology, 13(3), 362–374. 10.1177/0022002182013003007

[bibr12-00332941241231210] Boroson B. (2011). Autism spectrum disorders in the mainstream classroom: How to reach and teach students with ASDs. Scholastic Teaching Resources.

[bibr13-00332941241231210] Brehm J. W. Wright R. A. Solomon S. Silka L. Greenberg J. (1983). Perceived difficulty, energization, and the magnitude of goal valence. Journal of Experimental Social Psychology, 19(1), 21–48. 10.1016/0022-1031(83)90003-3

[bibr14-00332941241231210] Chaiken S. Eagly A. H. (1983). Communication modality as a determinant of persuasion: The role of communicator salience. Journal of Personality and Social Psychology, 45(2), 241–256. 10.1037//0022-3514.45.2.241

[bibr15-00332941241231210] Chartrand T. L. van Baaren R. B. Bargh J. A. (2006). Linking automatic evaluation to mood and information processing style: Consequences for experienced affect, impression formation, and stereotyping. Journal of Experimental Psychology: General, 135(1), 70–77. 10.1037/0096-3445.135.1.70 16478316 PMC2791521

[bibr16-00332941241231210] Chen S. Chaiken S. (1999). The heuristic-systematic model in its broader context. In Chaiken S. Trope Y. (Eds.), Dual-process theories in social psychology (pp. 73–96). The Guilford Press.

[bibr17-00332941241231210] Cohen J. Cohen P. West S. G. Aiken L. S. (2003). Applied multiple regression/correlation analysis for the behavioral sciences (3rd ed.). Lawrence Erlbaum Associates Publishers.

[bibr18-00332941241231210] Cohen L. D. Yavin L. L. Rubinsten O. (2021). Females’ negative affective valence to math related words. Acta Psychologica, 217, 1–11. 10.1016/j.actpsy.2021.103313 33930625

[bibr19-00332941241231210] Connolly F. White P. (2017). Game changer: The art of sports science. Victory Belt.

[bibr20-00332941241231210] Cury F. Elliot A. J. Da Fonseca D. Moller A. C. (2006). The social-cognitive model of achievement motivation and the 2 x 2 achievement goal framework. Journal of Personality and Social Psychology, 90(4), 666–679. 10.1037/0022-3514.90.4.666 16649862

[bibr21-00332941241231210] Dewhurst S. A. Anderson R. J. Knott L. M. (2012). A gender difference in the false recall of negative words: Women DRM more than men. Cognition & Emotion, 26(1), 65–74. 10.1080/02699931.2011.553037 21432635

[bibr22-00332941241231210] Dickens W. J. Perry R. P. (1982). Perceived control in college classrooms: The impact of student and teacher characteristics. [Paper presentation]. International Congress of Applied Psychology, Edinburgh, Scotland, 15–31 July, 1982.

[bibr23-00332941241231210] Doyle S. (2005). The manager’s pocket guide to motivating employees. HRD Press.

[bibr24-00332941241231210] DuBrin A. J. (2018). Tolerating ambiguity for leadership and professional effectiveness. Page Publishing Inc.

[bibr26-00332941241231210] Erez A. Isen A. M. (2002). The influence of positive affect on the components of expectancy motivation. Journal of Applied Psychology, 87(6), 1055–1067. 10.1037/0021-9010.87.6.1055 12558213

[bibr27-00332941241231210] Forsyth D. R. McMillan J. H. (1981). Attributions, affect, and expectations: A test of Weiner’s three-dimensional model. Journal of Educational Psychology, 73(3), 393–403. 10.1037/0022-0663.73.3.393

[bibr28-00332941241231210] Gesser-Edelsburg A. Walter N. Shir-Raz Y. Green M. S. (2015). Voluntary or mandatory? The valence framing effect of attitudes regarding HPV vaccination. Journal of Health Communication, 20(11), 1287–1293. 10.1080/10810730.2015.1018642 26132725

[bibr84-00332941241231210] Gist M. E. Mitchell T. R. (1992). Self-efficacy: A theoretical analysis of its determinants and malleability. The Academy of Management Review, 17, 183–211.

[bibr29-00332941241231210] Grensing-Pophal L. (1999). The HR book: Human resources management for small business. International Self-Counsel Press Ltd.

[bibr30-00332941241231210] Guadagno R. E. Cialdini R. B. (2007). Persuade him by email, but see her in person: Online persuasion revisited. Computers in Human Behavior, 23(2), 999–1015. 10.1016/j.chb.2005.08.006

[bibr31-00332941241231210] Hansten R. I. Jackson M. (2009). Clinical delegation skills: A handbook for professional practice (4th ed.). Jones and Bartlett Publishers.

[bibr32-00332941241231210] Hayes A. F. (2018). Introduction to mediation, moderation, and conditional process analysis (2nd ed.). The Guilford Press.

[bibr33-00332941241231210] Hill H. Ott F. Herbert C. Weisbrod M. (2006). Response execution in lexical decision tasks obscures sex-specific lateralization effects in language processing: Evidence from event-related potential measures during word reading. Cerebral Cortex, 16(7), 978–989. 10.1093/cercor/bhj040 16177269

[bibr34-00332941241231210] Hong Y.-y. Chiu C.-y. Dweck C. S. Lin D. M.-S. Wan W. (1999). Implicit theories, attributions, and coping: A meaning system approach. Journal of Personality and Social Psychology, 77(3), 588–599. 10.1037//0022-3514.77.3.588

[bibr35-00332941241231210] Hu X. Chen Y. Tian B. (2016). Feeling better about self after receiving negative feedback: When the sense that ability can be improved is activated. The Journal of psychology, 150(1), 72–87. 10.1080/00223980.2015.1004299 25699420

[bibr36-00332941241231210] Huang S.-c. Jin L. Zhang Y. (2017). Step by step: Sub-goals as a source of motivation. Organizational Behavior and Human Decision Processes, 141, 1–15. 10.1016/j.obhdp.2017.05.001

[bibr37-00332941241231210] Interview Skills Workbook , (n.d.). https://www.studocu.com/en-au/document/university-of-technology-sydney/early-interventions-in-acute-care-nursing/2021interviewskills-a4/36432078

[bibr38-00332941241231210] Jankowska A. Zielińska U. (2015). Designing a self-assessment instrument for developing the speaking skill at the advanced level. In Pawlak M. WaniekKlimczak E. (Eds.), Issues in teaching, learning and testing speaking in a Second language (pp. 251–265). Springer.

[bibr39-00332941241231210] Jordens K. Van Overwalle F. (2005). Cognitive dissonance and affect: An initial test of a connectionist account. Psychologica Belgica, 45(3), 157–184. 10.5334/pb-45-3-157

[bibr40-00332941241231210] Kandola R. Wood R. Dholakia B. Keane C. (2001). The graduate recruitment manual. Gower Publishing Limited.

[bibr41-00332941241231210] Karademas E. C. Kafetsios K. Sideridis G. D. (2007). Optimism, self-efficacy and information processing of threat- and well-being-related stimuli. Stress and Health, 23(5), 285–294. 10.1002/smi.1147

[bibr42-00332941241231210] Klein H. J. Wright P. M. (1994). Antecedents of goal commitment: An empirical examination of personal and situational factors. Journal of Applied Social Psychology, 24(2), 95–114. 10.1111/j.1559-1816.1994.tb00560.x

[bibr85-00332941241231210] Komarraju M. Nadler D. (2013). Self-efficacy and academic achievement: Why do implicit beliefs, goals, and effort regulation matter? Learning and Individual Differences, 25, 67–72.

[bibr43-00332941241231210] Kovenklioglu G. Greenhaus J. H. (1978). Causal attributions, expectations, and task performance. Journal of Applied Psychology, 63(6), 698–705. 10.1037//0021-9010.63.6.698

[bibr44-00332941241231210] Labroo A. A. Kim S. (2009). The “instrumentality” heuristic: Why metacognitive difficulty is desirable during goal pursuit. Psychological Science, 20(1), 127–134. 10.1111/j.1467-9280.2008.02264.x 19152545

[bibr45-00332941241231210] Lee R. Everett C. A. (2004). The integrative family therapy supervisor: A primer. Brunner-Routledge.

[bibr46-00332941241231210] Lewis L. M. Dember W. N. Schefft B. K. Radenhausen R. A. (1995). Can experimentally induced mood affect optimism and pessimism scores? Current Psychology, 14(1), 29–41. 10.1007/bf02686871

[bibr47-00332941241231210] Lewis S. K. Ross C. E. Mirowsky J. (1999). Establishing a sense of personal control in the transition to adulthood. Social Forces, 77(4), 1573–1599. 10.2307/3005887

[bibr48-00332941241231210] Life at United World . (2013). Right language drives workplace positivity. https://lifeatunitedworld.wordpress.com/2013/02/04/right-languagedrives-workplace-positivity/

[bibr49-00332941241231210] Logan T. K. Royse D. (2010). Program evaluation studies. In Thyer B. (Ed.), The handbook of social work research methods (2nd ed., pp. 221–240). Sage.

[bibr50-00332941241231210] Maxwell S. E. Delaney H. D. Kelley K. (2017). Designing experiments and analyzing data: A model comparison perspective (3rd ed.). Routledge.

[bibr51-00332941241231210] McAuley E. Duncan T. E. Russell D. W. (1992). Measuring causal attributions: The revised causal dimension scale (CDSII). Personality and Social Psychology Bulletin, 18(5), 566–573. 10.1177/0146167292185006

[bibr52-00332941241231210] McMahan I. D. (1973). Relationships between causal attributions and expectancy of success. Journal of Personality and Social Psychology, 28(1), 108–114. 10.1037/h0035474

[bibr53-00332941241231210] Michaelides M. P. Durkee P. (2021). Self-regulation versus self-discipline in predicting achievement: A replication study with secondary data. Frontiers in Education, 6, 724711. 10.3389/feduc.2021.724711

[bibr54-00332941241231210] Miele D. B. Molden D. C. (2010). Naive theories of intelligence and the role of processing fluency in perceived comprehension. Journal of Experimental Psychology: General, 139(3), 535–557. 10.1037/a0019745 20677898

[bibr55-00332941241231210] Myers C. G. Staats B. R. Gino F. (2014). ‘My bad!’ How internal attribution and ambiguity of responsibility affect learning from failure. Harvard Business School Working Paper Series, 1–53.

[bibr56-00332941241231210] Pahomov L. (2014). Authentic learning in the digital age: Engaging students through inquiry. ASCD.

[bibr57-00332941241231210] Peer E. Brandimarte L. Samat S. Acquisti A. (2017). Beyond the Turk: Alternative platforms for crowdsourcing behavioral research. Journal of Experimental Social Psychology, 70, 153–163. 10.1016/j.jesp.2017.01.006

[bibr11-00332941241231210] Prototype Training Systems (n.d.). Your “weaknesses” are areas of opportunity. https://prototypetraining.com/your-weaknesses-are-areas-of-opportunity/

[bibr58-00332941241231210] Rael R. (2012). Strategy and risk management. American Institute of Certified Public Accountants Inc.

[bibr59-00332941241231210] Ripp P. (2012). Let’s discuss your weaknesses and watch you soar. https://pernillesripp.com/2012/05/29/lets-discuss-your-weaknesses-and-watch-yousoar/

[bibr60-00332941241231210] Rudisill M. E. (1988). The influence of causal dimension orientations and perceived competence on adult’s expectations, persistence, performance, and the selection of causal dimensions. International Journal of Sport Psychology, 19(3), 184–198.

[bibr61-00332941241231210] Shieh G. (2019). Effect size, statistical power, and sample size for assessing interactions between categorical and continuous variables. British Journal of Mathematical and Statistical Psychology, 72(1), 136–154. 10.1111/bmsp.12147 30468259

[bibr62-00332941241231210] Silver W. S. Mitchell T. R. Gist M. E. (1995). Responses to successful and unsuccessful performance: The moderating effect of self-efficacy on the relationship between performance and attributions. Organizational Behavior and Human Decision Processes, 62(3), 286–299. 10.1006/obhd.1995.1051

[bibr63-00332941241231210] Simoni J. M. Adelman H. S. Nelson P. (1991). Perceived control, causality, expectations and help-seeking behaviour. Counselling Psychology Quarterly, 4(1), 37–44. 10.1080/09515079108254427

[bibr64-00332941241231210] Sjåstad H. Baumeister R. F. Ent M. R. (2020). Greener grass or sour grapes? How people value future goals after initial failure. Journal of Experimental Social Psychology, 88, 103965. 10.1016/j.jesp.2020.103965

[bibr65-00332941241231210] Sniezek J. A. (1999). Issues in self-control theory and research confidence, doubt, expectancy bias, and opposing forces. In Wyer Jr R. S. (Ed.), Perspectives on 45 behavioral self-regulation: Advances in social cognition (Vol. 12, pp. 217–228). Lawrence Erlbaum Associates Publishers.

[bibr66-00332941241231210] Spinella A. (2020). 2020 NBA draft: How valuable is the craftiness of Tre Jones? https://bballwriters.com/nba-draft/2020-nba-draft-how-valuable-isthe-craftiness-of-tre-jones/

[bibr86-00332941241231210] Staw B. M. (1976). Knee-deep in the Big Muddy: A study of escalating commitment to a chosen course of action. Organizational Behavior & Human Performance, 16, 27–44.

[bibr67-00332941241231210] Strunk K. K. Bailey L. E. (2015). The difference one word makes: Imagining sexual orientation in graduate school application essays. Psychology of Sexual Orientation and Gender Diversity, 2(4), 456–462. 10.1037/sgd0000136

[bibr68-00332941241231210] Sullivan L. (2018). The journey toward a sustainable, site-specific well-being education subject. In Perspectives on flourishing in schools (pp. 229–235). Lexington Books.

[bibr69-00332941241231210] Sweeney P. D. Moreland R. L. Gruber K. L. (1982). Gender differences in performance attributions: Students’ explanations for personal success or failure. Sex Roles, 8(4), 359–373. 10.1007/bf00287275

[bibr70-00332941241231210] Tafarodi R. W. Milne A. B. (2002). Decomposing global self-esteem. Journal of Personality, 70(4), 443–483. 10.1111/1467-6494.05017 12095187

[bibr71-00332941241231210] Tapper J. (2014). Gain an edge at job interviews. Xlibris LLC.

[bibr72-00332941241231210] Tolin P. (1970). Instruction effects on watch keeping in a “simple” vigilance task. Perception & Psychophysics, 9(2), 227–228. 10.3758/bf03212637

[bibr73-00332941241231210] Usher E. L. Pajares F. (2008). Self-efficacy for self-regulated learning: A validation study. Educational and Psychological Measurement, 68(3), 443–463. 10.1177/0013164407308475

[bibr74-00332941241231210] Violanti M. T. Kelly S. (2023). Self-assessments: Creating validated teaching and training tools. Business and Professional Communication Quarterly, 1–21.

[bibr75-00332941241231210] Vohs K. D. Baumeister R. F. (2004). Understanding self-regulation: An introduction. In Baumeister R. F. Vohs K. D. (Eds.), Handbook of self-regulation: Research, theory, and applications (pp. 1–9). The Guilford Press.

[bibr76-00332941241231210] Watson D. Clark L. A. Tellegen A. (1988). Development and validation of brief measures of positive and negative affect: The PANAS scales. Journal of Personality and Social Psychology, 54(6), 1063–1070. 10.1037//0022-3514.54.6.1063 3397865

[bibr77-00332941241231210] Weiner B. (1985). An attributional theory of achievement motivation and emotion. Psychological Review, 92(4), 548–573. 10.1037//0033-295x.92.4.548 3903815

[bibr78-00332941241231210] Weiner B. (2012). An attributional theory of motivation and emotion. Springer Science & Business Media.

[bibr79-00332941241231210] Wicker F. W. Hamman D. Reed J. H. McCann E. J. Turner J. E. (2005). Goal orientation, goal difficulty, and incentive values of academic goals. Psychological Reports, 96(3 Pt 1), 681–689. 10.2466/pr0.96.3.681-689 16050622

[bibr80-00332941241231210] Wigfield A. Eccles J. S. (2000). Expectancy–value theory of achievement motivation. Contemporary Educational Psychology, 25(1), 68–81. 10.1006/ceps.1999.1015 10620382

[bibr81-00332941241231210] Wilson T. D. Wheatley T. P. Kurtz J. L. Dunn E. W. Gilbert D. T. (2004). When to fire: Anticipatory versus postevent reconstrual of uncontrollable events. Personality and Social Psychology Bulletin, 30(3), 340–351. 10.1177/0146167203256974 15510418

[bibr82-00332941241231210] Zimmerman B. J. (2002). Becoming a self-regulated learner: An overview. Theory Into Practice, 41(2), 64–70. 10.1207/s15430421tip4102_2

[bibr83-00332941241231210] Zimmerman B. J. Kitsantas A. (2014). Comparing students’ self-discipline and selfregulation measures and their prediction of academic achievement. Contemporary Educational Psychology, 39(2), 145–155.

