# Supplementary material for: Should We Really Be Afraid of “Weakness”? Applying the Insights of Attribution Theory
Source: Psychol Rep. 2024 Feb 27;129(1):372–404. doi: 10.1177/00332941241231210 (PMC12717299; doi:10.1177/00332941241231210)
Supplement: Supplemental Material - Should We Really Be Afraid of “Weakness”? Applying the Insights of Attribution Theory [file sj-pdf-1-prx-10.1177_00332941241231210.pdf]

Online Appendices

Supplementary Analyses for Experiment 2

Confidence intervals (CIs) for (moderated) indirect effects of “weakness” on negative affect through perceived controllability and improvement expectancy, or through perceived internality) included zero. However, there was evidence of a negative indirect effect of “weakness” on positive affect through reduced PC and IE in sequence (i.e. “Weakness” → reduced perceived controllability → reduced improvement expectancy → reduced positive affect). The index of moderated moderated mediation (.10) was associated with a CI excluding zero [.0030,.1940]. The index of moderated mediation for men (-.03) was associated with a CI including zero, but the corresponding index for women (.06) was associated with a CI excluding zero [.0003, .1364]. Thus, amongst women the negative indirect effect of “weakness” on positive affect (PA) apparently depended on perceived selfefficacy for self-regulation (PSESR). Amongst women, when PSESR was low (49.93%), the negative effect of “weakness” on positive affect (through reduced perceived controllability and improvement expectancy) was estimated to be -1.91[-3.7269, -.4288] - a small effect. When PSESR was at the sample mean, this effect was estimated to be -1.01[-1.9461, -.2399] and when PSESR was high (79.15%), the estimated effect was negligible and not statistically different from zero: -.04[-1.0483,.9487].

For the effect of “weakness” on positive affect through increased perceived internality, the index of moderated moderated mediation (-.06) was also associated with a CI excluding zero [-.1199, -.0062]. The index of moderated mediation for women (-0.2) was associated with a CI including zero but the corresponding index for men (0.3) was associated with a CI excluding zero [.0007,.0776]. Thus, amongst men, the negative effect of

"weakness" on positive affect (through increased perceived internality) apparently depend on perceived self-efficacy for self-regulation (PSESR). Amongst men, when PSESR was low, this negative effect was estimated to be -.65[-1.5698,-.0380] - a very small effect. At moderate and high levels of PSESR the effect was estimated to be smaller still and CIs included zero
